# BMI trajectories among children diagnosed with type 1 diabetes mellitus at a tertiary diabetes center

**DOI:** 10.3389/fendo.2025.1537860

**Published:** 2025-03-17

**Authors:** Reem Abdullah Al Khalifah, Noor Salem Bawahab, Raghad Wadea, Hala Gasim, Alhanouf Alrashed, Muneera Al-Jelaify, Bayan Alnassir, Eman Ragab Saleh, Iman Al-Gadi

**Affiliations:** ^1^ Pediatric Endocrine Division, Department of Pediatrics, College of Medicine, King Saud University, Riyadh, Saudi Arabia; ^2^ The University Diabetes Centre, King Saud University Medical City, Riyadh, Saudi Arabia; ^3^ Pharmacy Services, King Saud University Medical City, Riyadh, Saudi Arabia

**Keywords:** type 1 diabetes mellitus, obesity, pediatric, Saudi Arabia (KSA), BMI - body mass index

## Abstract

**Objective:**

The incidence of type 1 diabetes mellitus (T1DM) has increased worldwide, raising concerns about the intersection between T1DM and the rising prevalence of childhood obesity. This study investigates secular trends in body mass index (BMI) at T1DM diagnosis and its trajectory post-diagnosis, focusing on predictors of obesity persistence.

**Methods:**

This retrospective cohort study was conducted at a tertiary diabetes center in Riyadh, Saudi Arabia, between January 2015 and December 2023. Children under 14 years diagnosed with T1DM at the center were included, while those diagnosed elsewhere or with other diabetes types were excluded. Data included demographics, BMI z-scores based on Saudi and CDC growth charts, HbA1c levels, and clinical presentations like diabetic ketoacidosis (DKA). Linear regression assessed secular trends and predictors of baseline BMI z-scores.

**Results:**

Among 1160 screened children’s charts, 408(35.17%) children met inclusion criteria. At diagnosis, mean age was 7.87 ± 3.53 years, with 161 (39.5%) presenting with DKA. The mean baseline BMI was 16.06 ± 3.62 kg/m², and BMI z-scores were -0.22 ± 1.65 (Saudi growth references) and -0.87 ± 1.92 (CDC growth references). No significant secular trends in BMI z-scores, gender, or DKA presentation were observed. Of 59(14.46%) children with baseline overweight/obesity, 46 (77.97%) remained overweight/obese at follow-up (p<0.001). The mean BMI z-score increased to 1.14± 1.64 at follow-up, with 113(27.70%) meeting overweight/obesity criteria.

**Conclusion:**

Overweight/obesity at diagnosis with T1DM persists at follow-up. Addressing obesity at T1DM diagnosis is crucial for mitigating its long-term metabolic impact. Future research should target interventions addressing lifestyle factors contributing to obesity in children with T1DM.

## Introduction

The global incidence of type 1 diabetes mellitus (T1DM) has been steadily rising, with an estimated rate of 36.9 cases per 100,000 individuals annually (1). The incidence of T1DM among children and adolescents in Saudi Arabia ranks among the highest globally, as reported in the latest International Diabetes Federation (IDF) Atlas. Moreover, Saudi Arabia is among the top five countries in terms of the prevalence of T1DM in children and adolescents, reflecting the growing burden of autoimmune diabetes in the region. Several studies have highlighted a rising trend in childhood T1DM cases over the past two decades, paralleling trends observed in other high-income and Gulf countries. This increase in T1DM incidence is accompanied by growing concern about the rising prevalence of overweight and obesity among children with T1DM, which may further complicate disease management and long-term outcomes (2).

Recent studies suggest a complex relationship between obesity and T1DM, where both the increasing prevalence of obesity and the autoimmune destruction of pancreatic beta cells intersect, possibly contributing to the rising incidence of T1DM in children (3). Globally, the prevalence of pediatric obesity has also seen a marked increase over the past three decades (4). In Saudi Arabia, childhood overweight/obesity rates have risen rapidly to 20.6%, aligning with trends observed worldwide (5). This trend is concerning as obesity, even at the time of diagnosis, may influence the disease’s clinical presentation and outcomes, including metabolic control and the risk of diabetic ketoacidosis (DKA).

However, gaps remain in understanding the secular trends of obesity among children diagnosed with T1DM and its possible impact on metabolic control. Therefore, we aim to investigate secular trends in BMI at the time of T1DM diagnosis over the past nine years, assess BMI trajectory following diagnosis, and identify potential predictors of BMI changes.

## Methods

### Study design

We conducted a retrospective cohort study at the University Diabetes Center at King Saud University Medical City (KSUMC), Riyadh, Saudi Arabia. The center is a large tertiary referral facility that accepts children from across Saudi Arabia. Typically, primary healthcare centers provide initial screening and referrals for suspected T1DM cases, which are referred for long-term management at specialized tertiary centers like KSUMC, ensuring comprehensive diagnosis, insulin therapy initiation, and multidisciplinary diabetes care.

The cohort included children aged less than 14 years newly diagnosed with T1DM at our center. In Saudi Arabia, pediatric endocrinology services typically manage T1DM patients until the age of 14 years, after which the care is transitioned to adult care. The data were collected between January 2015 and December 2023. This study period was chosen due to the availability of the Electronic Health System, and it assesses the secular trends over a meaningful timeframe while ensuring that changes observed are not influenced by short-term variations in clinical practice, healthcare policies, or the COVID-19 pandemic. Children who were diagnosed with T1DM at other centers were excluded due to the lack of baseline data, as well those with other types of diabetes.

We identified cases using the Electronic Health System of the Medical Records employing the International Classification of Diseases (ICD) ICD-9 and ICD-10 codes for ketoacidosis, type 1 diabetes, and hyperglycemia, in addition to all patients who received any form of insulin treatment in Emergency department.

### Measures

We reviewed the electronic medical records for age at diagnosis with T1DM, sex, weight, height, BMI, sex- and age-adjusted weight, height and z-score were calculated based on the Saudi and Centers for Disease Control and Prevention (CDC) growth chart, HbA1c levels at diagnosis and last follow-up visit, presence of diabetes ketoacidosis (DKA) at diagnosis, follow up duration, type of insulin therapy (6, 7).

Type 1 Diabetes Mellitus (T1DM) was defined using the International Society for Pediatric and Adolescent Diabetes (ISPAD) guidelines (8). According to the ISPAD, diabetes is diagnosed when there is hyperglycemia (random plasma glucose ≥11.1 mmol/L or fasting plasma glucose ≥7.0 mmol/L) accompanied by symptoms of hyperglycemia, such as polyuria, polydipsia, and weight loss. Pediatric obesity was defined using the CDC guidelines. According to the CDC, obesity in children is classified based on age- and sex-specific BMI percentiles derived from growth charts. Children with a BMI at or above the 95th percentile for their age and sex are considered obese, while those with a BMI between the 85th and 94th percentiles are classified as overweight (9, 10). While the CDC growth charts are widely used for international comparisons, their applicability to Middle Eastern populations remains debated due to regional differences in growth patterns. The Saudi growth charts provide population-specific references; however, they have not been updated in recent years. Given the secular changes in childhood growth patterns, our study findings may need to be interpreted within the context of evolving BMI distributions in the Saudi pediatric population (6, 7).

### Outcomes

The primary outcome of our study was to investigate secular trends in BMI z-scores at the time of diagnosis of T1DM. The secondary outcomes included: predictors of Baseline BMI z-scores, BMI trajectory post-diagnosis, prevalence of overweight/obesity, and the metabolic control: during the last clinic follow-up visit.

### Ethical considerations

We obtained approval for this study from the King Saud University Institutional Review Board. The study procedures complied with the Good Clinical Practice and Declaration of Helsinki.

### Statistical analysis

We reported baseline characteristics and clinical variables as means with standard deviations (SD) for continuous variables following a normal distribution, and as frequencies with percentages for categorical variables. To assess differences in continuous variables, we employed one-way ANOVA with Bonferroni adjustment for multiple comparisons. For categorical variables, we utilized the chi-square test. Additionally, we performed one-sample t-tests to compare cohort baseline BMI z-score and the known Saudi children’s values (6, 7).

To evaluate secular trends in BMI z-scores at diagnosis over the study period (2015–2023), we conducted linear regression analyses with BMI z-score as the dependent variable and year of diagnosis as the independent variable. We also examined the impact of diabetic ketoacidosis (DKA), gender, and age groups on baseline BMI z-scores using linear regression models. Missing baseline data were imputed using the group mean; for other missing values, the last observation carried forward (LOCF) method was applied. Longitudinal data analyses were performed to assess the influence of baseline BMI on follow-up BMI measurements. All statistical analyses were conducted using Stata/SE 16.0 for Mac.

## Results

Out of the initial cohort of 1,160 children, 408(35.17%) children were diagnosed at our center with T1DM, the remaining were excluded because 582(50.17%) were diagnosed with T1DM outside our center and do not have baseline at diagnosis data; 15(1.3%) had Type 2 Diabetes Mellitus; 14(1.2%) had neonatal diabetes mellitus; 11(1.0%) had maturity-onset diabetes of the young; 2(0.17) had cystic fibrosis related diabetes; 108(9.31%) were not cases with diabetes; 18 (1.55%) were over 14 years old at the time of diabetes diagnosis; and 2(0.17%) had chronic kidney disease.

**Figure 1:**
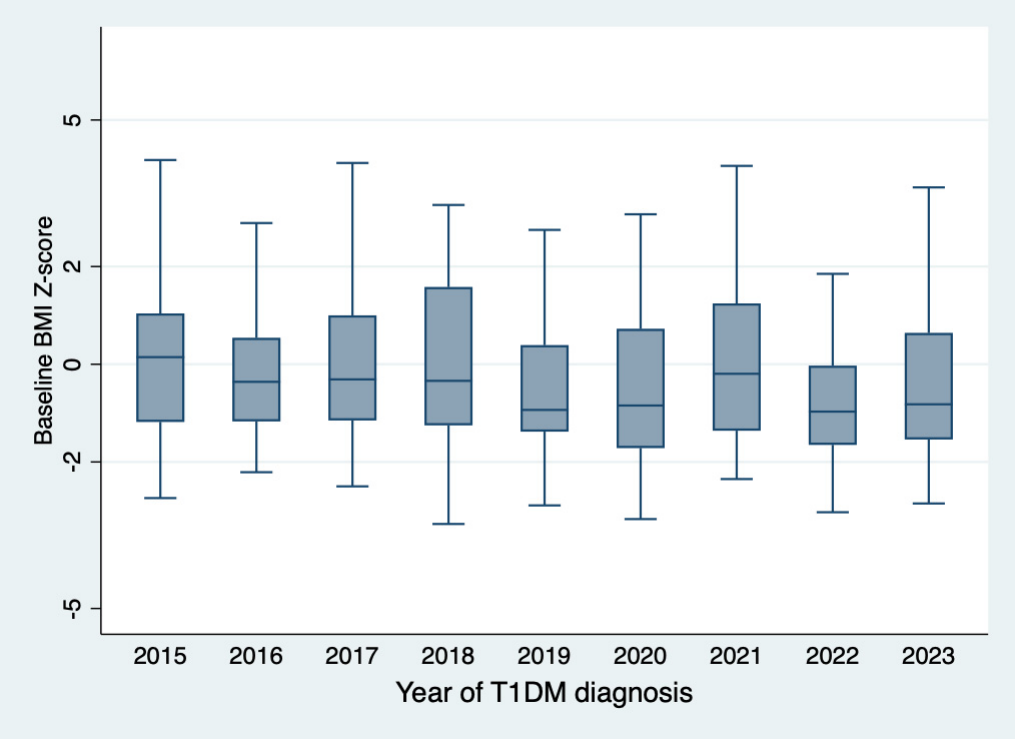
Baseline BMI z-score based on the Saudi normative data for children and adolescents.

The mean age of children presenting with new-onset T1DM at our center was 7.95 ± 3.50 years, with 209(51.23%) being male and 161(39.5%) presenting with DKA. The mean baseline BMI was 16.06 ± 3.62 kg/m². The BMI z-scores were -0.22 ± 1.65 SD based on Saudi growth charts and -0.87 ± 1.92 SD according to the CDC growth charts (**Table 1**). The observed difference between the Saudi Z-score and CDC Z-score is 0.66 ± 0.97. **Figure 1** illustrates the distribution of BMI z-scores across different age groups over the years of diagnosis. No significant secular trends were observed concerning the year of diagnosis, gender, or DKA status (**Table 2**). At baseline, 59(14.46%) children met the criteria for overweight or obesity. The mean cohort BMI z-score was comparable to that of Saudi children.

**Table 1:** Baseline characteristics and follow up outcomes at last appointment.

	Years of diagnosis with T1DM										P-value
	Overall	2015	2016	2017	2018	2019	2020	2021	2022	2023	
Number	408	34	57	50	43	39	40	53	46	46	
Age, years	7.95 (3.50)	8.19 (3.49)	7.86 (3.54)	8.08 (3.56)	8.57 (3.14)	7.58 (3.81)	7.34 (3.30)	8.04 (3.61)	7.05 (3.67)	8.81 (3.27)	0.33
Age category											
0-5 *	135 (33.10)	13 (9.63)	20 (14.81)	15 (11.11)	11 (8.15)	16 (11.85)	13 (9.63)	19 (14.07)	18 (13.33)	10 (7.41)	0.33
6-10 *	140 (34.31)	8 (5.71)	20 (14.29)	21 (15.0)	17 (12.14)	8 (5.71)	16 (11.43)	15 (10.71)	17 (12.14)	18 (12.86)	
11-14 *	133 (32.60)	13 (9.77)	17 (12.78)	14 (10.53)	15 (11.28)	15 (11.28)	11 (8.27)	19 (14.29)	11 (8.27)	18 (13.53)	
Sex, male*	209 (51.23)	12 (5.74)	28 (13.4)	23 (11.0)	24 (11.48)	21 (10.05)	22 (10.53)	32 (15.31)	22 (10.53)	25 (11.96)	0.54
DKA*	161 (39.50)	14 (8.7)	22 (13.66)	15 (9.32)	15 (9.32)	15 (9.32)	19 (11.8)	19 (11.8)	22 (13.66)	20 (12.42)	0.74
HbA1c, %	11.25(2.41)	10.89(1.66)	10.95(2.07)	11.44(2.32)	11.69(2.66)	11.19(2.52)	11.25(2.10)	11.89(2.98)	10.78(2.61)	11.04(2.29)	0.33
Weight, kg	27.81(13.42)	28.26(11.08)	26.57(12.46)	29.98(16.07)	30.43(13.20)	26.14(11.19)	25.84(14.10)	29.39(15.05)	24.01(12.55)	29.29(12.86)	0.27
Height, cm	127.93(22.21)	131.54()	125.14(22.03)	129.25(24.04)	132.70(19.27)	127.55(22.94)	124.23(20.85)	128.61(22.40)	121.30(25.20)	132.26(21.42)	0.20
Height z-score	0.41 (1.15)	0.30 (1.08)	0.09 (1.13)	0.58 (1.04)	0.59 (1.06)	0.51 (1.34)	0.62 (1.16)	0.56 (1.14)	0.19 (1.37)	0.33 (1.05)	0.20
BMI, kg/m^2^	16.06(3.62)	16.50(3.61)	16.01(3.17)	16.55(3.61)	16.40(3.54)	15.34(2.84)	15.95(4.70)	16.54(3.48)	15.11(3.02)	16.07(4.39)	0.48
BMI, CDC z-score	-0.87(1.92)	-0.67(2.08)	-0.78(1.59)	-0.47(1.68)	-0.77(2.07)	-1.35(1.96)	-1.30(2.24)	-0.46(1.71)	-1.31(2.11)	-0.94(1.95)	0.16
BMI, Saudi z-score	-0.22(1.65)	0.04 (1.69)	-0.20(1.46)	0.04 (1.63)	-0.05(1.70)	-0.55(1.39)	-0.37(2.00)	0.02 (1.60)	-0.67(1.50)	-0.27(1.87)	0.34
Overweight/Obesity *	59 (14.46)	3 (8.82)	8 (14.04)	8 (16)	8 (18.6)	3 (7.69)	6 (15)	10 (18.87)	5 (10.87)	8 (17.39)	0.79
Follow up at last clinic appointment											
Follow up duration, years	4.11 (2.55)	7.31 (2.68)	6.17 (2.70)	5.78 (1.87)	4.89 (1.68)	4.03 (1.35)	3.52 (1.00)	2.90 (0.70)	1.72 (0.64)	0.86 (0.45)	<0.001
Lost to follow up *	61 (15.02)	8 (23.53)	17 (30.36)	9 (18)	11 (25.58)	5 (12.82)	3 (7.69)	3 (5.66)	2 (4.35)	3 (6.52)	<0.001
Multiple daily injection *	393 (96.32)	33 (97.06)	53 (92.98)	47 (94)	41 (95.31)	36 (92.31)	39 (97.50)	53 (100)	46 (100)	45 (97.83)	0.67
Follow up HbA1c, %	9.24 (1.99)	9.66 (2.57)	9.79 (2.03)	9.32 (1.56)	9.72 (2.23)	9.62 (2.08)	9.04 (1.64)	8.87 (1.84)	8.86 (1.88)	8.18 (1.72)	0.008
Weight, kg	45.75(20.50)	56.53(16.13)	49.71(17.97)	54.29(19.69)	53.63(23.96)	44.61(18.32)	42.86(20.97)	44.46(21.72)	31.23(14.71)	35.39(14.80)	<0.001
Hight, cm	145.39(30.79)	155.75(13.09)	151.46(16.51)	153.80(16.75)	151.72(17.69)	146.32(22.71)	141.87(20.80)	142.29(21.27)	130.35(22.52)	136.38(20.39)	<0.001
Height z-score	-0.33(1.04)	-0.70(0.92)	-0.78(1.12)	-0.46(1.08)	-0.42(0.99)	-0.20(0.91)	-0.21(1.06)	-0.05(0.97)	-0.09(0.92)	-0.02(1.16)	<0.001
BMI, kg/m^2^	20.32(5.01)	22.65(5.56)	20.99(4.56)	22.16(5.23)	22.07(6.41)	19.62(3.77)	19.87(5.22)	20.61(5.17)	17.22(3.03)	17.82(3.44)	<0.001
BMI, CDC z-score	0.31 (1.29)	0.51 (1.39)	0.20 (1.31)	0.39 (1.70)	0.42 (1.44)	0.20 (1.03)	0.41 (1.00)	0.72 (1.13)	-0.09(1.07)	0.01 (1.25)	0.08
Overweight/Obesity *	113(27.70)	12 (35.29)	17 (29.82)	16 (32)	16 (37.21)	7 (17.95)	9 (22.5)	21 (39.62)	7 (15.22)	8 (17.39)	0.05

Values are mean± SD unless indicated.

*N (%).

**Table 2:** Univariate regression model for the impact on baseline BMI Z-score.

Model	Coefficient	95% Confidence Interval	P-value	Adjusted R-squared
DKA	-0.19	-0.58, 0.20	0.33	0.00
Gender, male	0.20	-0.17, 0.58	0.30	0.00
Year of Diagnosis	-0.05	-0.12, 0.234	0.18	0.00

**Figure 2:**
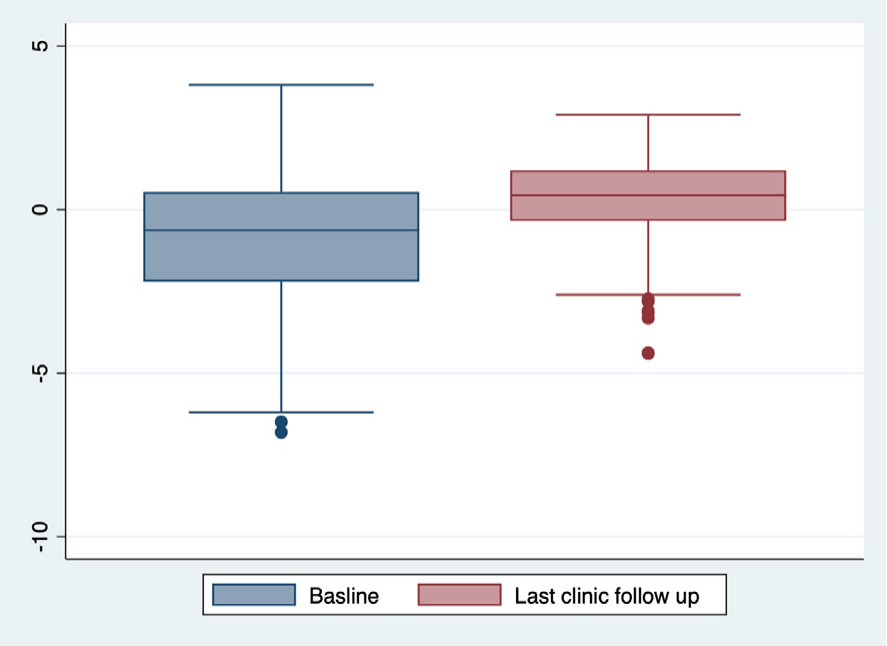
BMI Z-score progression from baseline to last clinic follow up.

The cohort had a mean follow-up duration of 4.11 ± 2.55 years, with 61(15.02%) children lost to follow-up due to transfers to other hospitals. At the final clinic visit, the mean BMI z-score was 0.31 ± 1.29 SD, and 113(27.7%) children were classified as overweight or obese (**Table 1**, **Figure 2**). No significant secular trends were observed concerning the year of diagnosis and final clinic visit mean BMI z-score, even when adjusted for follow-up duration. Notably, the mean BMI z-score increased at follow up by 1.14± 1.64. Children who were overweight or obese at baseline tended to remain in the same category at follow-up; of the 59(14.46%) children initially overweight or obese, 46 (77.97%) remained so at the last clinic visit (p < 0.001) (**Table 3**). This pattern was consistent across all age groups.

**Table 3:** Transitional status of obesity between baseline and last clinic follow up.

Age Group	Remined Normal	Developed Obesity	Became Normal	Remained Obese	P-value
0-5 years	104/124 (83.87%)	20/124 (16.13%)	1/11 (9.09%)	10/11 (90.91%)	<0.001
6-10 years	97/119 (81.51%)	22/119 (18.49%)	6/21 (28.57%)	15/21 (71.43%)	<0.001
11-14 years	81/106 (76.42%)	25/106 (23.58%)	6/27 (22.22%)	21/27 (77.78%)	<0.001

## Discussion

The global prevalence of overweight and obesity has risen alarmingly, representing a critical public health concern (4). Our study addresses a critical gap in understanding the interplay between obesity and T1DM in children, particularly in Saudi Arabia, where childhood obesity rates have reached 20.6%. Contrary to expectations, our findings revealed no secular trends in obesity prevalence or BMI z-score trajectory across age groups over the past nine years (11). Although yearly fluctuations in obesity prevalence were observed, these changes were not statistically significant. Gender, age, and presentation with DKA also had no discernible impact on baseline BMI z-scores. Additionally, no difference was observed between the baseline BMI z-scores of our cohort and the general Saudi pediatric population.

Notably, the baseline prevalence of obesity in our cohort was lower than that reported in international T1DM cohorts. For instance, the SEARCH for diabetes in youth study reported an obesity prevalence of 22%, while the Joslin diabetes center cohort reported a prevalence of 31% (12, 13). Similar trends have been observed in adult cohorts, with obesity rates of 22% in Kuwait, 5% in Mexico, and 12% in Belgium (14). These variations may reflect differences in study design, population demographics, healthcare systems, and cultural or dietary factors.

The relatively lower prevalence in our cohort underscores the importance of considering regional and methodological differences when interpreting obesity trends in children with T1DM. Additionally, the lower BMI z-score at baseline may reflect the weight loss commonly observed prior to insulin initiation (15). The increase in BMI following T1DM diagnosis is multifactorial, with insulin therapy playing a well-established role in weight gain. The anabolic effects of insulin contribute to increased adiposity by promoting lipogenesis and inhibiting lipolysis, particularly in the early post-diagnosis period when insulin therapy is initiated to counteract pre-diagnosis weight loss (16). Furthermore, insulin dose escalation to achieve glycemic control may be associated with increased appetite and reduced energy expenditure. The impact of different insulin regimens, such as multiple daily injections (MDI) versus continuous subcutaneous insulin infusion (CSII), should also be considered, as CSII therapy has been linked to greater BMI increases in pediatric populations compared to MDI. Future studies should explore strategies to mitigate insulin-induced weight gain, such as optimizing basal-bolus insulin regimens, adjunctive therapies targeting insulin sensitivity, and structured nutritional and physical activity interventions (17).

The persistence of obesity among children with T1DM underscores the importance of integrating obesity management into diabetes care at diagnosis (18). In our cohort, majority of children classified as overweight or obese at baseline remained in the same category at follow-up, paralleling global trends (15, 18). Importantly, the prevalence of overweight/obesity at follow-up increased to 27.70%, aligning more closely with international observations. This underscores the need for clinicians to routinely monitor BMI, incorporate dietary counseling, and encourage physical activity in diabetes management plans (19).

Our study utilized both the Saudi and CDC growth charts to assess BMI z-scores, highlighting the population-specific differences in growth patterns. The Saudi growth charts, published in 2007, reflect regional growth trajectories but may not fully capture recent secular trends in pediatric BMI (20). In contrast, the CDC growth charts (2000) provide an internationally recognized standard but may not be directly applicable to Saudi children due to genetic, nutritional, and environmental differences. The discrepancies in BMI z-scores between the two references highlight fundamental population differences in growth patterns.

Our study also highlights the positive impact of multidisciplinary care and continuous glucose monitoring on improving metabolic outcomes and patient retention. However, it is important to note that our findings are derived from a single tertiary center, which may limit their generalizability to broader populations. While our study spanned nine years, the sample size remains a limiting factor in detecting subtle secular trends in BMI. Future multi-center studies with larger sample sizes are needed to confirm the presence of regional or temporal shifts in BMI at T1DM diagnosis. A notable limitation of our study is the loss to follow-up in 15% of the cohort, which may have introduced selection bias. Factors contributing to attrition likely include hospital transfer and variations in adherence to follow-up care. Studies have shown that children from lower socioeconomic backgrounds and those with poorly controlled diabetes are at higher risk of being lost to follow-up. Understanding the determinants of follow-up discontinuation may help develop strategies to improve patient retention and long-term monitoring of BMI trajectories (21). Additionally, annual trends in BMI, HbA1c, dietary intake, physical activity, and sleep patterns, and other metabolic parameters were not analyzed. Future research should explore these factors and develop targeted interventions to mitigate obesity’s impact on metabolic outcomes in children with T1DM.

In conclusion, while our cohort demonstrated lower obesity prevalence at diagnosis compared to other international T1DM cohorts, the persistence of obesity post-diagnosis calls for comprehensive strategies to address obesity alongside T1DM management.

## Data Availability

The raw data supporting the conclusions of this article will be made available by the authors, without undue reservation.
